# Optical silencing of body wall muscles induces pumping inhibition in *Caenorhabditis elegans*

**DOI:** 10.1371/journal.pgen.1007134

**Published:** 2017-12-27

**Authors:** Megumi Takahashi, Shin Takagi

**Affiliations:** Division of Biological Science, Nagoya University Graduate School of Science Chikusa-ku, Nagoya, Japan; Brown University, UNITED STATES

## Abstract

Feeding, a vital behavior in animals, is modulated depending on internal and external factors. In the nematode *Caenorhabditis elegans*, the feeding organ called the pharynx ingests food by pumping driven by the pharyngeal muscles. Here we report that optical silencing of the body wall muscles, which drive the locomotory movement of worms, affects pumping. In worms expressing the Arch proton pump or the ACR2 anion channel in the body wall muscle cells, the pumping rate decreases after activation of Arch or ACR2 with light illumination, and recovers gradually after terminating illumination. Pumping was similarly inhibited by illumination in locomotion-defective mutants carrying Arch, suggesting that perturbation of locomotory movement is not critical for pumping inhibition. Analysis of mutants and cell ablation experiments showed that the signals mediating the pumping inhibition response triggered by activation of Arch with weak light are transferred mainly through two pathways: one involving gap junction-dependent mechanisms through pharyngeal I1 neurons, which mediate fast signals, and the other involving dense-core vesicle-dependent mechanisms, which mediate slow signals. Activation of Arch with strong light inhibited pumping strongly in a manner that does not rely on either gap junction-dependent or dense-core vesicle-dependent mechanisms. Our study revealed a new aspect of the neural and neuroendocrine controls of pumping initiated from the body wall muscles.

## Author summary

Since feeding is an essential behavior for the survival of animals, it is modulated by a variety of neural and neuroendocrine signals that are generated depending on internal and external conditions. To elucidate the cellular and molecular mechanisms underlying the regulation of feeding, the nematode *Caenorhabditis elegans*, which is composed of a small number of identifiable cells, provides a unique system. In *C*. *elegans*, the pumping movement of a feeding organ called the pharynx has been subjected to intensive genetic studies. Compared to the factors promoting pumping, however, the inhibitory mechanisms of pumping are less well understood. In this paper, we report that optogenetic silencing of the body wall muscles, which drive the locomotory movement of worms, inhibits pumping in the pharynx. Signals emanating from muscles are likely to trigger pumping inhibition, raising an interesting possibility that the proprioceptive sense detecting the relaxation of body wall muscles might be involved. When the Arch proton pump was activated with weak light, signals for pumping inhibition are transferred into the pharynx mainly through two pathways: one involving gap junction-dependent mechanisms through pharyngeal I1 neurons, which mediate fast signals, and the other involving dense-core vesicle-dependent mechanisms, which mediate slow signals. Strong activation of Arch inhibits pumping very strongly via other mechanisms. Thus, we have revealed a new link between pumping and the body wall muscles, and confirmed the important cooperation of neural and neuroendocrine circuits in the regulation of feeding behaviors.

## Introduction

Feeding is essential for the survival, growth, and proliferation of animals. Accordingly, feeding behaviors change depending on both the internal physiological state and external environmental conditions [1]. For studying the neural and neuroendocrine mechanisms underlying the control of feeding behaviors, the nematode *Caenorhabditis elegans*, which is amenable to a variety of experimental manipulations, provides a unique system.

*C*. *elegans* takes food in through a hollow organ called the pharynx that is located in the head [2]. Morphologically, the pharynx consists of three parts: the corpus, isthmus, and terminal bulb (TB), in anterior to posterior order. The pharyngeal muscles in the corpus and the TB undergo cycles of synchronized contraction and relaxation [3]. This rhythmic movement, called pumping, allows the worm to swallow and filter foods such as *Escherichia Coli* in liquid and expel excess fluid. The pharynx also contains the pharyngeal nervous system that comprises 20 neurons of 14 types. Electron microscopy (EM) study showed that the pharynx is isolated from the rest of the body by a basement membrane, and the synaptic connection between the pharyngeal and the extrapharyngeal nervous systems is limited to a gap junction between the I1 neuron in the pharynx and the RIP neuron outside the pharynx [4].

The pharyngeal nervous system participates in the regulation of pumping. Remarkably, the pharynx can continue pumping even when all of the pharyngeal neurons are ablated, although the frequency of pumping decreases profoundly [5]. MC pharyngeal neurons are mainly responsible for high-frequency pumping; ablation of the other pharyngeal neurons did not affect pumping frequency significantly [6]. These results indicate that pumping of the pharynx is basically myogenic, similar to pumping of the vertebrate heart [7], with the pharyngeal nervous system playing mainly modulatory roles. The pharynx, however, stops pumping under certain conditions. Pumping ceases during lethargus, the period before molting [8]. Various types of stress, such as heat shock [9][10], physical stimulation to the body [11][12], and blue light [13][14], induce pumping quiescence. Thus, neural mechanisms that repress the spontaneous activity of the pharyngeal muscles must exist. The I1-RIP connection is, in fact, required for pumping inhibition caused by tail tap [15], though the details remain unknown.

Previous studies have also shown that the neuroendocrine system, including biogenic amines such as serotonin [16][17][18] and peptides [10] secreted from dense-core vesicles, is involved in the regulation of the pumping rate. Although the pumping rate decreases without food in wild-type (WT) animals, the rate remains high in *unc-31* mutants [19], which are defective for post-docking calcium-regulated dense-core vesicle fusion. Pumping inhibition by exposure to a high level of CO_2_ is also impaired in *unc-31* mutants [20]. A neuropeptide FLP-13 expressed in the extrapharyngeal ALA neuron mediates pumping inhibition after heat shock [10]. Although these findings underscore the importance of the neuroendocrine control of pumping, the manner in which peptides secreted by extrapharyngeal cells affect the pumping remains poorly understood.

Optogenetic tools have been widely used for controlling the activity of neurons and muscle cells by using light illumination *in vivo*. Archaerhodopsin-3(Arch) is a light-driven outward proton pump [21], frequently used in *C*. *elegans* for silencing muscle activity [22][23][24]. Here we report that optical silencing of the body wall muscles induces reduction in the pumping rate. Our genetic studies revealed that two independent pathways, one employing neural transmission and the other employing neurohumoral signals, are involved in the transmission of signals for the pumping inhibition response.

## Results

### Silencing of the body wall muscles induced pumping inhibition in *C*. *elegans*

**Fig 1:**
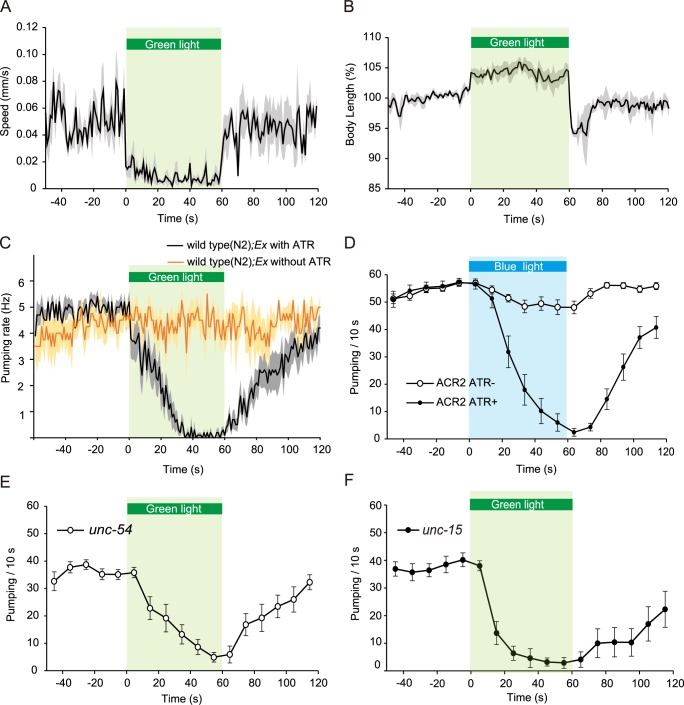
Silencing of the body wall muscles caused pumping inhibition in addition to reduction in locomotion speed and elongation of the body length in *C*. *elegans*. (A) Illumination with green light (22 mW/mm^2^) reduced moving speed in an ST300: *Ex(myo-3p*::*Arch*::*gfp*, *rol-6d)* animal whose body wall muscles expressed Arch. The speed dropped immediately after starting illumination with green light, and the locomotion stopped almost completely during the light illumination (1 min, green bar). Immediately after the light was turned off, the locomotion resumed (n = 5 worms) In all legends, if the number of trials is unspecified, each worm was assayed once). The black line indicates the average moving speed, and the gray area indicates standard error of the mean (SEM). (B) The change in body length during the trials is shown in (A). The body length was normalized so that the average body length during the 1 min period before starting illumination was 100%. The black line indicates the average length, and the gray area indicates SEM. The body was elongated by approximately 5% during illumination for 1 min. For approximately 10 s after the light was turned off, the body length became shorter than it was before illumination was initiated. Approximately 20 s after the light was turned off, the body length returned to the level prior to illumination. (C) Pumping rate in ST300 *Ex(myo-3p*::*Arch*::*gfp*, *rol-6d)* animals. The rate was gradually decreased by the green light (22 mW/mm^2^), and 30 s after illumination was initiated, the pumping stopped almost completely. Black line: worms cultivated with all-*trans*-retinal (ATR) (n = 11 worms), orange line: worms without ATR for control (n = 4 worms). The green light did not affect the movement, body length, or pumping rate of the worms raised without ATR. (D) Pumping rate in ST371*Ex(myo-3p*::*ACR2*::*gfp*,*rol-6d)* animals. The rate was gradually decreased by the blue light (1.5 mW/mm^2^), and it recovered gradually after the light was turned off. Black line with filled circles: worms cultivated with all-*trans*-retinal (ATR) (n = 19 worms), black line with open circles: worms without ATR for control (n = 16 worms). (E) Pumping rate of ST311 *unc-54(e190); ncEx3031(myo-3p*::*Arch*::*gfp*, *rol-6d)* animals cultivated with ATR (n = 11 worms) modulated by green light illumination (5.5 mW/mm^2^). Pumping number for each 10 s period was plotted in the graph. Error bars indicate ± standard error of the mean (SEM). (F) Pumping rate of ST363 *unc-15(e73); ncIs53(myo-3p*::*Arch*::*gfp)* animals cultivated with ATR (n = 11 worms) modulated by green light illumination (5.5 mW/mm^2^). Pumping number for each 10 s period was plotted in the graph. Error bars indicate ± SEM.

In a previous report, we expressed Archaerhodopsin-3(Arch), a green light-driven proton pump, in the body wall muscle cells of worms using the *myo-3* promoter carried by an extrachromosomal array, *ncEx3031*. The transgenic animal, ST300, ceased forward and backward movement immediately after starting illumination with green light (550 nm) [22] (Fig 1A and 1B). At the same time, the body of the animal became straighter and longer. The arrest of the locomotory movement and body elongation were sustained throughout the 1 min period of illumination and, after illumination was stopped, the animal immediately recovered normal locomotory movement and body length. In addition, we noticed that illumination affected pharyngeal pumping. This unexpected finding prompted us to further examine pumping both during and after illumination.

We recorded pumping over a period of 3 min, which spans before (1 min), during (1 min), and after (1 min) illumination (Fig 1C). Green light illumination at 22 mW/mm^2^ gradually decreased the pumping rate, and pumping stopped completely 30 s after illumination was initiated. The pumping rate in animals that were grown without all-*trans*-retinal (ATR) as a control did not change at all upon illumination (Fig 1C). The pumping remained inhibited for a while after the light was turned off, in sharp contrast to the quick recovery of the locomotory movement and body length. The time lag between the response of the body to illumination and that of pumping suggests that the pumping inhibition is not the result of the silencing of the pharyngeal muscles by green light, but is instead a consequence of the silencing of the body wall muscles.

Previous studies have shown that the *myo-3* gene encodes a myosin heavy chain expressed specifically in the body wall muscles and vulva muscles, but not in the pharyngeal muscles whereas the *myo-2* gene, encoding another myosin heavy chain protein, is expressed specifically in the pharyngeal muscles. To confirm that Arch::GFP driven by the *myo-3* promoter is not expressed in the pharyngeal muscles, we generated a transgenic line, ST326, carrying *myo-2p*::*mCherry* together with *myo-3p*::*Arch*::*gfp*. (S1A and S1B Fig)[25][26]. A fluorescence micrograph of ST326 showed that the GFP and mCherry signals did not merge in the pharyngeal muscles (S1C Fig). This indicates that green light did not silence the pharyngeal muscles directly in animals carrying *myo-3p*::*Arch*::*gfp*.

These results suggested that silencing the body wall muscles is the key event that triggers pumping inhibition. It is, however, possible that light activation of the Arch proton pump might have effects other than silencing cells. For example, it may lower the extracellular pH, which can consequently activate neighboring cells expressing acid-sensing ion channels (ASICs) [27]. It is also possible that hyperpolarization beyond the physiological level might have unknown pathological effects. To circumvent these problems, we attempted to silence the body wall muscles using ACR2, a natural light-gated anion channel [28][29]. In the transgenic line ST371 expressing ACR2::GFP by the *myo-3* promoter, we found that blue light illumination at 1.5 mW/mm^2^, which immediately inhibited the locomotion of the animals, also caused pumping inhibition in a manner similar to that caused by Arch (Fig 1D).

The light illumination induced two apparent changes in the body: body elongation and arrest of locomotion. To test whether unperturbed forward movement is necessary for maintaining pumping at a high frequency, we examined pumping in mutants with locomotion defects. UNC-54 is a myosin heavy chain expressed in the body wall muscles, but not in the pharyngeal muscles. The *unc-54 (e190*) mutants rarely moved [30], but the pumping rate was not significantly different from that in WT animals. We found that illumination with green light in *unc-54* mutants carrying *myo-3p*::*Arch*::*gfp* decreased the pumping rate (Fig 1E). The *unc-15 (e73)* mutants, whose paramyosin gene is affected [31], exhibit a locomotion-defective phenotype similar to that of the *unc-54* mutants. We found that light activation of *myo-3p*::*Arch* in the *unc-15* mutants also led to pumping inhibition (Fig 1F). Thus, hindered locomotion itself is not required for inducing pumping inhibition. Moreover, illumination induced elongation of the body in the *unc-54* mutant background; the body lengthened by 1.5 ± 0.6% 0.5 s after illumination with green light was initiated (n = 4 worms). These observations imply that elongation of the body is correlated with pumping inhibition.

### Pumping inhibition was affected by certain defects in neurotransmission

**Fig 2:**
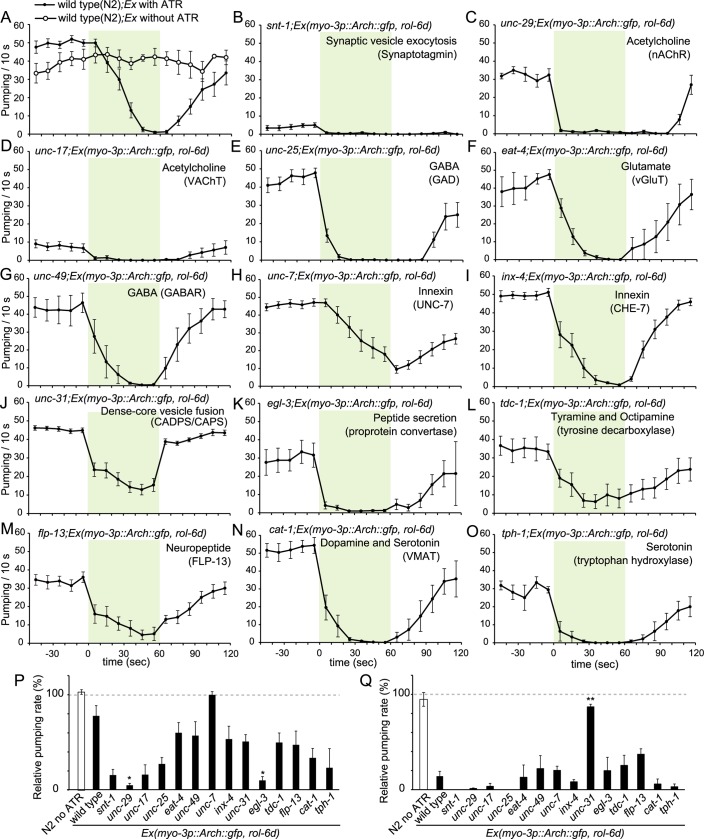
Modulation of pharyngeal pumping caused by silencing of the body wall muscles from illumination with green light (22 mW/mm^2^) in ST300 *Ex(myo-3p*::*Arch*::*gfp*, *rol-6d)* animals carrying mutations in genes involved in neurotransmission. (A) The black line with filled circles and the dashed line with open circles indicate the pumping number in each 10 s time frame in ST300 *Ex(myo-3p*::*Arch*::*gfp*, *rol-6d)* animals cultivated in the presence and absence of ATR, respectively. The data are shown in Fig 1C. Without ATR: n = 4 worms; with ATR: n = 10 worms. Error bars indicate the SEM. (B) Modulation of pharyngeal pumping in ST303 *snt-1(n2665); ncEx3031(myo-3p*::*Arch*::*gfp*, *rol-6d)* animals (n = 5 worms). (C) ST306 *unc-29(e193); ncEx3031* (n = 5 worms). (D) ST310 *unc-17(e245); ncEx3031* (n = 5 worms). (E) ST309 *unc-25(e156); ncEx3031* (n = 5 worms). (F) ST312 *eat-4(ad572); ncEx3031* (n = 5 worms). (G) ST308 *unc-49(e382); ncEx3031* (n = 5 worms). (H) ST314 *unc-7(e5); ncEx3031* (n = 10 worms). (I) ST315 *inx-4(e1128); ncEx3031* (n = 9 worms). (J) ST302 *unc-31(e928); ncEx3031* (n = 19 worms). (K) ST304 *egl-3(n729); ncEx3031]* (n = 5 worms). (L) ST319 *tdc-1(n3420); ncEx3031* (n = 8 worms). (M) ST320 *flp-13(tm2427); ncEx3031* (n = 10 worms). (N) ST313 *cat-1(e1111); cnEx3031* (n = 5 worms). (O) ST305 *tph-1(n4622); ncEx3031* (n = 5 worms). (P) Relative pumping rate in each mutant during the 10 s period immediately after illumination was initiated is shown. The pumping rate in each animal during the 10 s period immediately before illumination was started was set as the standard. Immediately after illumination was initiated, the pumping rate in *unc-7* mutants changed very little. Differences compared to ST300 were examined using the Steel's many-one rank sum test (*: *p* < 0.05). (Q) The relative pumping rate in each mutant during the 10 s period immediately after illumination was stopped is shown. The pumping rate during the 10 s period immediately before illumination was stopped was set as the standard for each animal. *unc-31* mutant animals started pumping immediately after illumination was stopped. Error bars indicate the SEM in all graphs. Differences compared to ST300 were examined using the Steel's many-one rank sum test (**: *p* < 0.01).

To reveal the neural mechanisms underlying the Arch-mediated pumping inhibition response, we examined various mutants defective in neural transmission (Fig 2). The number of pumps during the 10 s period immediately after starting illumination (Fig 2P) and immediately after stopping illumination (Fig 2Q) was counted in animals carrying *myo-3*p::*Arch*::*gfp* as an extrachromosomal array.

We first examined the involvement of major neurotransmitters, such as acetylcholine, glutamate, and GABA. Green light illumination induced pumping inhibition in mutants for genes encoding a vesicular acetylcholine transporter UNC-17/VAChT, a vesicular glutamate transporter EAT-4/vGluT, and a GABA-synthesizing enzyme UNC-25/GAD. Although the sample size is small, this indicated that none of the major classical neurotransmitters is sufficient on their own to fully convey information for the pumping inhibition.

It has been reported that the release of acetylcholine from MC neurons is important for the maintenance of high pumping rates [6]. As reported previously, in *snt-1/synaptotagmin* mutants defective for neurotransmitter release, the pumping rate is very low (Fig 2B) [32]. The rate decreased further during illumination, however. The *unc-17/VAChT* mutants defective for ACh release responded similarly to illumination (Fig 2D). These results suggest that the pumping inhibition is not completely caused by the reduction in acetylcholine release from MC neurons.

Although none of the mutations singly abolished the pumping inhibition response completely, two of them, *unc-7* and *unc-31*, which partly suppressed the pumping inhibition response in different manners, attracted our attention. UNC-7 is one of the innexin proteins forming gap junctions in invertebrates [33]. In *unc-7* mutants, the pumping rate decreased slowly during illumination, and also recovered slowly after illumination was stopped (Fig 2H and 2P). UNC-31 is *C*. *elegans* CADPS/CAPS required for the calcium-dependent secretion of dense-core vesicles [34]. In *unc-31* mutants, the pattern of the induction of pumping inhibition did not significantly differ from that in WT animals, but inhibition was incomplete during the 1 min period of illumination. After the green light was turned off, the pumping rate quickly returned to the normal level (Fig 2J and 2Q). These results indicated the distinct roles of UNC-7-dependent gap junctions and UNC-31-dependent secretion of dense-core vesicles in conveying signals mediating the pumping inhibition response triggered by Arch-mediated body wall muscle silencing.

### UNC-7-dependent and UNC-31-dependent pathways mediating pumping inhibition

**Fig 3:**
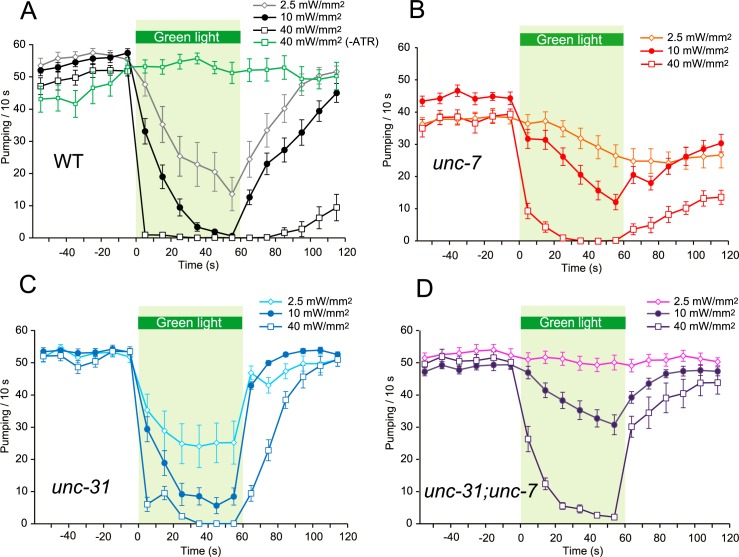
Modulation of the pumping rate in animals carrying the integrated transgene, *ncIs53(myo-3p*::*Arch*::*gfp*, *rig-3p*::*mCherry)*. Dependence of modulation of the pumping rate on intensity of green light illumination (A) Pumping rate in ST322 *ncIs53(myo-3p*::*Arch*::*gfp*, *rig-3p*::*mCherry)* animals when illuminated with green light at three different intensities: weak (2.5 mW/mm^2^), medium (10 mW/mm^2^), and strong (40 mW/mm^2^). 2.5 mW/mm^2^, n = 12 worms; 10 mW/mm^2^, n = 27 worms; 40 mW/mm^2^, n = 14 worms; 40 mW//mm^2^ without ATR, n = 6 worms. (B) Modulation of the pumping rate of ST362 *unc-7(e5); ncIs53* animals caused by illumination with green light at different intensities. 2.5 mW/mm^2^, n = 12 worms; 10 mW/mm^2^, n = 19 worms; 40 mW/mm^2^, n = 10 worms. (C) Modulation of the pumping rate in ST361 *unc-31(e928); ncIs53* animals caused by illumination with green light at different intensities. 2.5 mW/mm^2^, n = 14 worms; 10 mW/mm^2^, n = 21 worms; 40 mW/mm^2^, n = 12 worms. (D) Modulation of the pumping rate in ST325 *unc-31(e928); unc-7(e5); ncIs53* animals caused by illumination with green light at different intensities. 2.5 mW/mm^2^, n = 13 worms; 10 mW/mm^2^, n = 13 worms; 40 mW/mm^2^, n = 13 worms.

**Fig 4:**
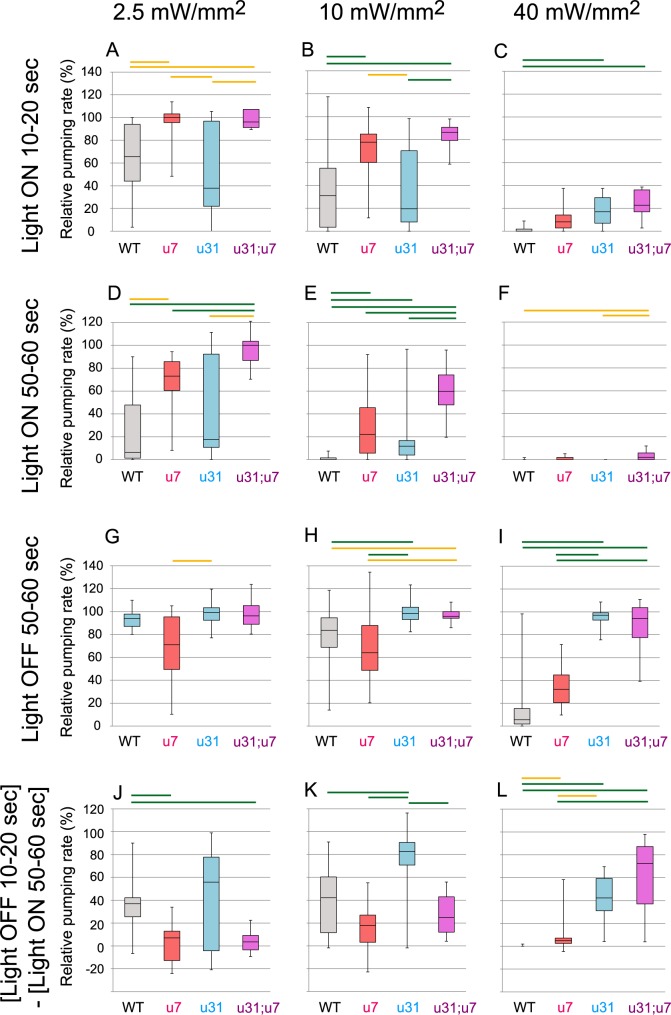
Comparison of modulation of the pumping rate among mutant animals carrying *ncIs53(myo-3p*::*Arch*::*gfp*, *rig-3p*::*mCherry)*. A box-whisker plot of the relative pumping rate during the 10 s period of WT, *unc-7(e5)*, *unc-31(e928)*, and *unc-31(e928); unc-7(e5)* animals shown in Fig 3. The boxes include 50% of the data. The inner line marks the median value, and whisker lines extending from the box represent the minimum and maximum values. The pumping rate during the 10 s period immediately before illumination was initiated was set as the standard for each animal. The relative pumping rate during the 10 s period between 10 and 20 s after illumination was initiated (A-C: Light ON 10–20 s), between 50 and 60 s after illumination was initiated (D-F: Light ON 50–60 s), and the 10 s period between 50 and 60 s after illumination was terminated (G-I: Light OFF 50–60 s) is shown. As a measure of a post-illumination recovery, the change between the relative pumping rate during the 10 s period between 10 and 20 s after illumination was turned off (light OFF 10–20 s), and the rate immediately before illumination was turned off (light ON 50–60 s) was also determined. J-L: (Light OFF 10–20 s)–(Light ON 50–60 s) for each animal. u7, u31, and u31;u7 indicates *unc-7(e5)*, *unc-31(e928)*, and *unc-31(e928); unc-7(e5)* animals, respectively. Green and yellow bars above the plots show the statistical significances of difference among strains determined by the Kruskal-Wallis test with post-hoc Steel-Dwass multiple comparison test (*p* < 0.01 and *p* < 0.05, respectively).

For further examination of the involvement of UNC-7 and UNC-31, we used *ncIs53* worms carrying chromosomally integrated *myo-3*::*Arch*::*gfp* transgenes, which showed a pumping inhibition response similar to, but more constant than, that with the extrachromosomal transgene *ncEx3031* used in the preceding sections. First, we examined the response to green light with an intensity of 10 mW/mm^2^ (Figs 3 and S3). Although almost all of the *ncIs53* worms stopped pumping 40 s after starting illumination (Fig 3A); a fraction of the *unc-7* (*e5*) mutants failed to stop pumping during the 1 min period of illumination (Figs 3B and 4E). The *unc-7* mutants also exhibited a retarded response to turning on/off the light compared to the WT. Namely, they continued high-frequency pumping for a while after starting green light illumination, and took a longer time to recover from inhibition after the light was turned off (Figs 3B and 4B, 4H and 4K). Another *unc-7* allele, *e139*, showed a similar phenotype (S4A Fig). In contrast, in the *unc-31* (*e928*) mutants, pumping inhibition was rapidly induced, similar to that in WT animals, and post-illumination recovery to the normal pumping rate was instantaneous (Figs 3C and 4B and 4K). Another *unc-31* allele, *e169*, showed a similar phenotype (S4B Fig). From these results, we speculated that the UNC-7-dependent pathway is responsible for triggering the rapid response whereas the UNC-31-dependent pathway mediates the slow and lingering response.

In order to examine the relationship between the UNC-7-dependent pathway and the UNC-31-dependent pathway, we generated *unc-31*(*e928*);*unc-7*(*e5*) double mutants. After 1 min illumination, the average reduction of pumping in the double mutants was limited by 40% (Figs 3D and 4E). This indicates that the UNC-7/innexin-dependent pathway and the UNC-31/CADPS/CAPS-dependent pathway play a major role in conveying signals for the pumping inhibition response caused by illumination of light with this intensity.

We then examined the dependence of the pumping inhibition response on the intensity of light. At a lower intensity of 2.5 mW/mm^2^, the pumping was also inhibited in WT, *unc-7*(*e5*), and *unc-31*(*e928*) animals, albeit to a lesser extent compared with that at 10 mW/mm^2^ (Figs 3A, 3B and 3C and 4D): pumping was reduced to 24%, 74%, and 46% on average, respectively, 1 min after initiating illumination. *unc-7*(*e5*) and *unc-31*(*e928*) animals exhibited a slow and rapid response to turning the light on/off, respectively (Figs 3B, 3C and 4A, 4J), similar to that with illumination at 10 mW/mm^2^. In contrast, pumping in *unc-31*(*e928*);*unc-7*(*e5*) double mutants was minimally affected (Figs 3D and 4D): the average reduction after illumination for 1 min was limited to 95%. This indicates that pumping-inhibiting signals caused by illumination with this intensity of light are mediated almost exclusively by the UNC-7-dependent and UNC-31-dependent pathways.

At a higher intensity of 40 mW/mm^2^, WT animals stopped pumping almost instantly when illumination was initiated, while the pumping rate in animals raised without ATR was not affected upon illumination (Fig 3A). *unc-7*(*e5*), *unc-31*(*e928*), and *unc-31*(*e928*); *unc-7*(*e5*) animals also exhibited a rapid reduction in pumping and stopped pumping almost completely 1 min after illumination was initiated (Figs 3B, 3C and 3D and 4F). This indicates that pumping inhibition with this intensity of light is mostly independent of both UNC-7 and UNC-31. Post-illumination recovery in *unc-31*(*e928*) and *unc-7*(*e5*):*unc-31*(*e928*) animals was rapid (Figs 3C, 3D and 4L) whereas pumping in WT and *unc-7*(*e5*) animals recovered only partly even 1 min after illumination was terminated (Figs 3A, 3B and 4I).

Taken together, these results support the notion that the UNC-7-dependent pathway mediates rapid signals, and the UNC-31-dependent pathway mediates slow signals, and that they function in parallel, at least in part, to inhibit pumping. At higher light intensity, there is a mechanism mediating a predominant inhibitory effect on pumping, which is independent of both UNC-7 and UNC-31.

### UNC-7-dependent inhibitory system transmits inhibitory signal via I1 neurons

**Fig 5:**
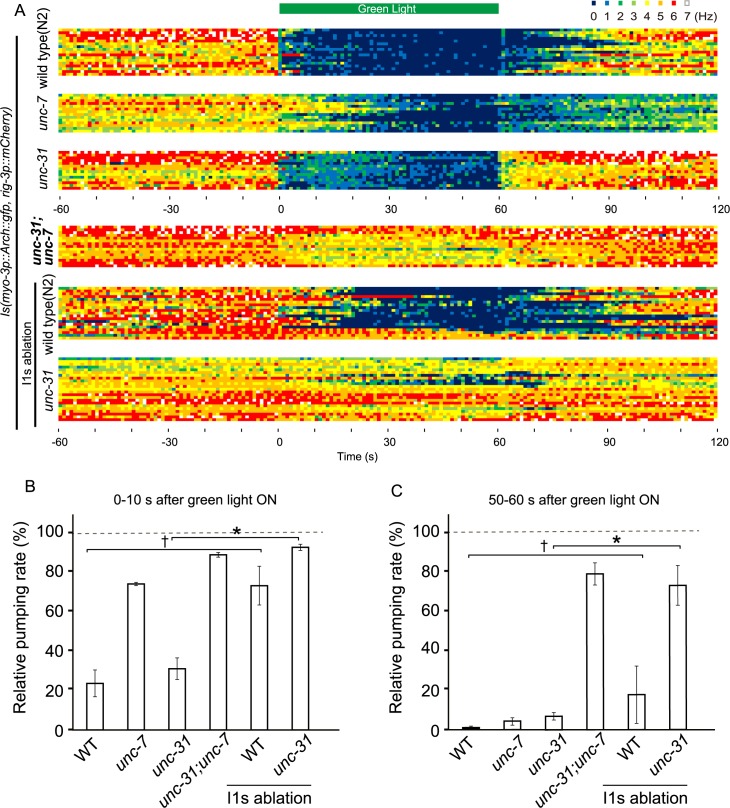
Effects of ablation of I1 neurons on modulation of the pumping rate by green light illumination at low intensity (A) The frequency of pumping before, during, and after light illumination (5.5 mW/mm^2^) represented by the heat map. Dark blue indicates no pumping, and red indicates six pumps/s as shown on the upper right. Each row indicates one trial. The time courses of the modulation of pumping in WT (N2), *unc-7(e5)* mutant, *unc-31(e928)* mutant, and *unc-31;unc-7* double mutant animals are shown. *unc-31;unc-7* double mutant animals continued pumping throughout the 1 min period of illumination. WT animals, in which the I1 neurons were ablated, exhibited a similar pattern of modulation to that in *unc-7* mutants. The pumping rate in *unc-31* animals in which the I1 neurons were ablated did not decrease during illumination, and was similar to that in *unc-31;unc-7* double mutants. WT (N2): n = 3 worms, 17 trials; *unc-7*: n = 3 worms, 14 trials; *unc-31*: n = 3 worms, 15 trials; *unc-31;unc-7*: n = 3 worms, 15 trials; WT (N2), I1 ablation: n = 4 worms, 19 trials; *unc-31*, I1 ablation: n = 5 worms, 23 trials. (B) Relative pumping rate during the 10 s period after illumination was started as calculated from the data shown in (A). The pumping rate during the 10 s period immediately before illumination with green light was used for normalization. The statistical significances of the difference among strains were determined by the Mann-Whitney U test (*:*p* < 0.05; †: *p* < 0.1). (C) Relative pumping rate during the 10 s period immediately before illumination was stopped as calculated from the data shown in (A). The pumping rate during the 10 s period immediately before illumination was set as the standard. The statistical significances of the difference among strains were determined by the Mann-Whitney U test. (*:*p* < 0.05; †: *p* < 0.1).

The gap junction formed between I1 and RIP is the sole neural connection linking the pharyngeal and extrapharyngeal nervous systems [4]. It was reported that UNC-7 is expressed in the pharyngeal I1 neurons [33]. To test the possibility that the UNC-7-dependent pathway requires I1 neurons for transmitting inhibition signals from the extrapharyngeal nervous system, we ablated the I1 neurons. We performed the experiment with green light with an intensity of 5.5 mW/mm, where the average reduction of pumping in *unc-7*(*e5*):*unc-31*(*e928*) animals after illumination for 1 min was approximately 20% (Figs 5C and S6). The pumping in the I1(-) worm showed retarded responses to turning on the green light, similar to those in the *unc-7* mutants compared to WT animals (*p* < 0.1. WT: n = 3; I1 (-): n = 4) (Fig 5A and 5B). In addition, we found that ablation of I1s in *unc-31(e928)* mutants exhibited a reduced pumping inhibition response: reduction in pumping after illumination for 1 min was, on average, 30% (Figs 5C and S6), which was comparable to that in the *unc-31*:*unc-7* double mutants, and differed from the control *unc-31* mutants (*p* < 0.05. *unc-31*: n = 3; *unc-31* I1 (-): n = 5). These results indicate that ablation of I1s and defects of UNC-7 have similar effects on the pumping inhibition response, suggesting that the UNC-7-dependent pathway transmits the signals via I1 neurons. These results also indicate that the UNC-31-dependent pathway functions independently, at least partly, from transmission via RIP-I1, which is the only neural connection between the intra- and extra-pharyngeal nervous systems.

## Discussion

Various stimuli are known to induce pumping inhibition in *C*. *elegans*. Here we have reported for the first time that the activation of optogenetic silencers in the body wall muscles affects pumping. This newly found phenomenon provides a unique opportunity to study the regulatory mechanisms of pumping that operate under free-moving conditions.

Our genetic screen revealed two major pathways mediating signals for pumping inhibition caused by Arch-mediated silencing of body wall muscles: the UNC-7/innexin-dependent pathway and the UNC-31/CADPS/CAPS-dependent pathway. This confirmed the important cooperation of neural and neuroendocrine circuits in the regulation of feeding behaviors.

### The cue for the induction of pumping inhibition

How does the activation of optogenetic silencers in the body wall muscle cells trigger the pumping inhibition response? We found that pumping was inhibited not only by activation of the Arch proton pump, but also by activation of the ACR2 anion channel, suggesting strongly that relaxation of the body wall muscle cells can in itself trigger the response. Our analyses using locomotion-defective mutants also showed that arrested movement caused by relaxation of the body wall muscle cells is not critical for the induction of pumping inhibition.

The cellular and molecular mechanisms underlying the presumed perception of the relaxation of the body wall muscle cells remain unclear. Muscle cells may emit some unknown retrograde signals in response to forced relaxation. Alternatively, changes in body posture caused by forced relaxation of the body wall muscles may play an important role. Interestingly, we found that pumping inhibition upon optical silencing of the body wall muscles, even in a locomotion-defective *unc-54* mutant, concurred with the body elongation. We speculate that there is a possible involvement of the proprioceptive sense detecting the status of muscle cells. Muscles in animals have various types of proprioceptive organs that detect changes in the tension and length of the muscles [35]. In *C*. *elegans*, DVA neurons and B-type motor neurons have been reported to sense body curvature in order to control the bending of the body [36][37]. Certain mechanoreceptors, which are usually involved in sensing touch stimuli, may also play a role in detecting changes in body posture. Whether these neurons are involved in the perception of muscle relaxation that triggers the pumping inhibition response remains to be determined in future studies.

Although our result using the ACR2 anion channel strongly suggests that body wall muscle relaxation causes pumping inhibition, it does not exclude the possibility that other factors are involved in pumping inhibition caused by activation of the Arch proton pump. We found that activation of Arch with illumination at a high intensity (40 mW/mm^2^) has a strong and quick inhibitory effect on pumping, which does not rely on the UNC-7/innexin-dependent and UNC-31/CADPS/CAPS-dependent pathways. Strong activation of Arch might lead to changes in certain physiological conditions in the body, such as pH, which could directly affect the pharynx. The possible involvement of factors other than body wall muscle relaxation in the Arch-mediated pumping inhibition remains to be examined in future studies. Further analyses of pumping inhibition using the ACR2 anion channel would also be helpful to clarify the issue.

### UNC-7-dependent pathway for inhibiting pumping

Our genetic experiments revealed that pathways mediating the pumping inhibition response partly depend on UNC-7/ innexins. UNC-7 is expressed in pharyngeal I1 and extrapharyngeal RIP neurons [33] to form gap junctions between them [4]. Since electrical synapses between I1-RIP are the only neural connection linking intra- and extra-pharyngeal neurons, it is likely that direct synaptic inputs to the pharynx are compromised in *unc-7* mutants. Our finding that I1-ablation affected the pumping inhibition in a manner similar to the *unc-7* mutation supports the notion that UNC-7 functions in I1s to transmit the pumping inhibition signal from RIPs into the pharynx.

A critical role of the electrical coupling of I1s and RIPs in the pumping inhibition response was previously suggested: Ivermectin, which is an agonist of the glutamate-gated chloride channel in *C*. *elegans*, kills worms through inhibition of feeding whereas *unc-7* mutants are resistant to this drug [38]. It has also been reported that pumping inhibition caused by a tail tap was partly repressed in *unc-7* mutants [12]. Recent studies have shown that optogenetic activation and silencing of I1 neurons induces an increase and decrease in the pumping rate, respectively [13][39]. Based on these facts, we speculate that relaxation of the body wall muscles is likely to result in hyperpolarization of I1s, which in turn induces the pumping inhibition response. It can also be speculated that the presumed hyperpolarization of I1s is transmitted from RIPs via gap junctions. The quick response of pumping to turning on/off the light in *unc-31* mutants is consistent with the idea that the UNC-7-dependent pathway transfers fast signals in which ordinary chemical and electrical synapses are engaged.

### UNC-31-dependent pathway for inhibiting pumping

Our genetic analysis using *unc-31* mutants indicates that pathways mediating the pumping inhibitory signal partly rely on a dense-core vesicle-dependent mechanism. In *unc-7* mutants, the pumping responded relatively slowly to turning the light on/off. These observations imply that the UNC-31-dependent pathway mediates humoral signals, which have slow and lasting effects; this is consistent with the known function of UNC-31/CAPS for secretion of neuropeptides and biological amines [34][40].

Previous studies reported the involvement of UNC-31 in pumping inhibition caused by different types of stimuli [19][41][42]. We found that the pumping inhibition by green light illumination occurs even when I1 neurons, the sole connectors between the extra- and intra-pharyngeal nervous systems, were ablated. Thus, in the UNC-31-dependent pathway, it is highly likely that factors secreted by extrapharyngeal tissues act directly on the pharynx. In fact, several neuropeptides that are secreted from extrapharyngeal neurons are known to affect pumping [43][44]. Notably, FLP-13 secreted by extrapharyngeal ALA neurons promotes quiescence, including repression of pumping following heat shock [10]. In our experiment, however, *flp-13* mutations failed to affect the pumping inhibition response significantly, suggesting the participation of other inhibitory factors.

### Pumping and postural status

Although *C*. *elegans* continues pumping almost throughout its lifetime, the pumping rate is modulated under certain circumstances that influence the posture of worms. For example, the swimming movement of worms in liquid consists of body movements that are quite distinct from crawling on solid agar. When crawling worms start swimming in water, they stop pumping [45]. When the body is stabilized physically, worms also stop pumping [39]. Both heat shock and lethargus arrest the body movement concomitantly with reducing the pumping rate [8][46][47]. Therefore, it seems that pumping and body posture are somehow linked. Although the physiological relevance of pumping inhibition caused by relaxation of the body wall muscle cells remains to be examined in the future, it may play a role in coordinating the pumping with the body posture through feedback from the latter to the former.

## Materials and methods

### *Caenorhabditis elegans* strains and maintenance

*C*. *elegans* strains were grown on a bacterial lawn of *E*. *coli* OP50 on nematode growth medium (NGM) agar [48]. Animals were maintained at 20°C. Plates with all-*trans*-retinal (ATR) (Sigma-Aldrich, St. Louis, USA) were kept in the dark.

### Plasmid construction

A destination vector containing the *rig-3* promoter, which drives gene expression in a subset of neurons including I1 neurons, pDEST-*rig-3p*, was constructed by inserting the polymerase chain reaction (PCR)-amplified genomic fragment into the *Sph*I site of pDEST-PL (a gift from Hidehito Kuroyanagi) using the following primers: 5’aaGCATGCggaaaaatgtgagatcttcgctgaaa3’ and 5’aaGCATGCgaatgaagttcttctgcaaggaatga3’. pGW-*rig-3p*::*mCherry* was generated by recombination between pDEST-*rig-3p* and pENTR-*mCherry* using the Gateway system (Invitrogen, San Diego, USA). pMT001: *myo-3p*::*ACR2*::*gfp* was generated by recombination between pDEST-*myo-3p* and pENTR-ACR2::gfp [29]. pOKA049: *myo-3p*::*Arch*::*gfp* was described previously [22].

### Transgenic strains

Transgenic animals were generated by microinjection of DNA into the gonad of N2 hermaphrodites [49]. pOKA049 (*myo-3p*::*Arch*::*gfp*, 100 ng/μl) and pGW-*rig-3p*::*mCherry* (50 ng/μl) were injected together into N2 worms to create strain ST357 carrying *ncEx9112*. pOKA049 (75 ng/μl), pRF4 (*rol-6d*, 125 ng/μl), and pCFJ90 (*myo-2p*::*mCherry*, 10 ng/μl) were injected together to create strain ST326 carrying *ncEx9198*. Strain ST371 carrying *ncEx3941* was generated by the injection of pMT001 (*myo-3p*::*ACR2*::*gfp*, 300 ng/μl) and pRF4 (*rol-6d*, 100 ng/μl) into N2 worms. Strain ST300 carrying *ncEx3031(myo-3p*::*Arch*::*gfp; rol-6d)* [22] was crossed with mutants to create the following strains with different mutant backgrounds:

ST302 *unc-31(e928) IV;ncEx3031(myo-3p*::*Arch*::*gfp*, *rol-6d)*, ST303 *snt-1(n2665) II;ncEx3031(myo-3p*::*Arch*::*gfp*, *rol-6d)*, ST304 *egl-3(n729) V;ncEx3031(myo-3p*::*Arch*::*gfp*, *rol-6d)*, ST305 *tph-1(n4622) II;ncEx3031(myo-3p*::*Arch*::*gfp*, *rol-6d)*, ST306 *unc-29(e193) I;ncEx3031(myo-3p*::*Arch*::*gfp*, *rol-6d)*, ST308 *unc-49(e382) III;ncEx3031(myo-3p*::*Arch*::*gfp*, *rol-6d)*, ST309 *unc-25(e156) III;ncEx3031(myo-3p*::*Arch*::*gfp*, *rol-6d)*, ST310 *unc-17(e245) IV;ncEx3031(myo-3p*::*Arch*::*gfp*, *rol-6d)*, ST311 *unc-54(e190) I;ncEx3031(myo-3p*::*Arch*::*gfp*, *rol-6d)*, ST312 *eat-4(ad572) III;ncEx3031(myo-3p*::*Arch*::*gfp*, *rol-6d)*, ST313 *cat-1(e1111) X;cnEx3031(myo-3p*::*Arch*::*gfp*, *rol-6d)*, ST314 *unc-7(e5) X;ncEx3031(myo-3p*::*Arch*::*gfp*, *rol-6d)*, ST315 *inx-4(e1128) V;ncEx3031(myo-3p*::*Arch*::*gfp*, *rol-6d)*, ST319 *tdc-1(n3420) II;ncEx3031(myo-3p*::*Arch*::*gfp*, *rol-6d)*, ST320 *flp-13(tm2427) IV;ncEx3031(myo-3p*::*Arch*::*gfp*, *rol-6d)*.

For chromosomal integration, ST357 L4 larvae were gamma-irradiated with cobalt 60 [50]. The integrated transgene strain ST322 carrying *ncIs53(myo-3p*::*Arch*::*gfp*, *rig-3p*::*mCherry)* was outcrossed seven times, and then crossed with *unc-7* and *unc-31* mutants to generate the following strains:

ST323 *unc-31(e928) IV; ncIs53(myo-3p*::*Arch*::*gfp*, *rig-3p*::*mCherry)*, ST324 *unc-7(e5) X; ncIs53(myo-3p*::*Arch*::*gfp*, *rig-3p*::*mCherry)*, ST325 *unc-31(e928) IV; unc-7(e5) X; ncIs53(myo-3p*::*Arch*::*gfp*, *rig-3p*::*mCherry)*, ST361 *unc-31(e169) IV; ncIs53(myo-3p*::*Arch*::*gfp*, *rig-3p*::*mCherry)*, ST362 *unc-7(e139) X; ncIs53(myo-3p*::*Arch*::*gfp*, *rig-3p*::*mCherry)*, ST363 *unc-15 (e73) I; ncIs53(myo-3p*::*Arch*::*gfp*, *rig-3p*::*mCherry)*.

### Behavioral assay under illumination

In the strains carrying the *Ex3031*(*myo-3p*:*Arch*::*gfp*, *rol-6d*) transgene, animals strongly expressing Arch::GFP were used for behavioral assays. Animals were moved onto experimental plates seeded with a solution of OP50 containing 500 μM ATR immediately before transfer of the animals, and incubated for at least 8 h before the assay was started.

The behavioral analyses were performed under an upright stereo-microscope (MVX10, Olympus) with an objective lens (MV PLAPO 2xC, Olympus). Movies were acquired at a rate of 30 frames/s with a digital video camera (GZ-HM570B, JVC) mounted on the microscope. For activation of Arch, animals were illuminated with a fluorescent light source (U-HGLGPS, Olympus) through a filter set (U-MRFPHQ/XL, OLYMPUS). Animals carrying the *myo-3p*:*ACR2*::*gfp* transgene were illuminated with a blue light (460–480 nm) through a filter set (U-MWIB2, OLYMPUS) at the intensity of 1.5 mW/mm^2^. At this intensity, the blue light by itself did not affect the pumping rate, as described previously [14]. The duration of light illumination was controlled with a shutter controller (SSH-C2B, Sigma Koki) using software (SSH-C2B_Demo_Software, Sigma Koki). Light intensity was measured as described previously [22].

### Measurement of pharyngeal pumping rates

Animals that pumped constantly were chosen for experiments. The pharyngeal pumping was counted manually by visual inspection of movies played at slow speed. One movement sequence consisting of an opening and a closing of the TB was counted as one pumping movement.

The basal pumping rate sometimes differed among mutants. In order to compare the responses to green light for different mutant backgrounds, the number of pumps during the 10 s period before green light illumination of the respective mutants was set as a standard for normalization of the pumping rate.

In assays of N2, *unc-31*, *unc-7*, and *unc-31;unc-7* double mutant animals, we excluded trials in which the pumping rate was less than 25 pumps per 10 s during the 30 s before light stimulation was initiated.

### Laser ablation of I1s

Laser ablations were performed using two-photon microscopy (ZEISS, LSM880; control soft: ZEN2 black edition; two-photon laser generation: Chameleon, COHERENT; objective lens: ZEISS Plan-APOCHROMAT 63× oil immersion lens). Animals were mounted on 4% agar pads and anesthetized with 10 mM levamisole. I1 neurons were identified in transgenic L1/L2 larvae expressing mCherry under the control of the *rig-3* promoter. The cell body of I1s was irradiated with an 860 nm laser (Bleaching Mode; Pixel Dwell: 8.24 μs; Iterations: 20; laser power: 100%). Ablation of I1s was confirmed by the absence of fluorescence in the cell body position. Behavioral assays were conducted on adult animals following a recovery period of 1 to 3 days after ablation. After the behavioral assay, the pharyngeal neurons in the animal were observed using confocal microscopy (FV300, Olympus) to ensure cell ablation.

### Measurement of body length and locomotion speed

The method used to measure animal body length was described previously [51]. The body length of ST311 *unc-54; ncEx3031(myo-3p*::*Arch*::*gfp*, *rol-6d)* was measured using the ImageJ public domain software and compared with that at 0.5 s before and after applying light illumination.

### Statistical analysis

The pumping rate in a 10 s period after illumination relative to that before starting illumination was calculated, and the scores were used for statistical analysis. The Steel's many-one rank sum test, Kruskal-Wallis test with post-hoc Steel-Dwass multiple comparison test and Mann-Whitney U Test was performed for data shown in Figs 2, 4, and 5, respectively. The statistical results are compiled in S1, S2 and S3 Tables, respectively.

## Supporting information

S1 Fig The *myo-3* promoter does not induce the expression of Arch in the pharyngeal muscles. (A) Expression of Arch::GFP driven by the *myo-3 promoter* in an adult ST300 worm carrying *Ex(myo-3p*::*Arch*::*gfp)*. The scale bar is 100 μm.
(B) A high magnification image of the white square frame in (A). Arch::GFP was expressed in the body wall muscles. The scale bar represents 20 μm.
(C) A fluorescent micrograph of an ST326 animal carrying *Ex(myo-3p*::*Arch*::*gfp*, *myo-2p*::*mCherry)*. mCherry was expressed in the pharyngeal muscles in which Arch::GFP was not expressed. The scale bar represents 50 μm.
(EPS)

S2 Fig Illumination with the green light caused elongation of the body in an ST311: *unc-54; Ex(myo-3p::Arch::gfp, rol-6d)* animal. The upper panel shows a worm 0.5 s before the light was turned on, and the bottom panel shows the same worm 0.5 s after the light was turned on. The left cross indicates the anterior tip of the head, and the right cross indicates the posterior tip of the tail of the animal before illumination was initiated. The scale bar is 100 μm.
(EPS)

S3 Fig A box-whisker plot of the relative pumping rate during a 10 s period in WT, *unc-7(e5), unc-31(e928)*, and *unc-31(e928); unc-7(e5)* animals during and after illumination with green light at different intensities (2.5 mW/mm2, 10 mW/mm2, and 40 mW/mm2) shown in Fig 3. The pumping rate during the 10 s period immediately before illumination was set as the standard. The boxes include 50% of the data. The inner line marks the median value, and whisker lines extending from the box represent the minimum and maximum values. A: WT, 2.5 mW/mm^2^, n = 12 worms; B: WT, 10 mW/mm^2^, n = 27 worms; C: WT, 40 mW/mm^2^, n = 14 worms; D: *unc-7(e5)*, 2.5 mW/mm^2^, n = 12 worms; E: *unc-7(e5)*, 10 mW/mm^2^, n = 19 worms; F: *unc-7(e5)*, 40 mW/mm^2^; G: *unc-31(e928)*, 2.5 mW/mm^2^, n = 14 worms; H: *unc-31(e928)*, 10 mW/mm^2^, n = 21 worms; I: *unc-31(e928)*, 40 mW/mm^2^, n = 12 worms; J: *unc-31(e928); unc-7(e5)*, 2.5 mW/mm^2^, n = 13 worms; K: *unc-31(e928); unc-7(e5)*, 10 mW/mm^2^, n = 13 worms; L: *unc-31(e928); unc-7(e5)*, 40 mW/mm^2^, n = 13 worms.
(EPS)

S4 Fig Modulation of the pumping rate by green light illumination (10 mW/mm^2^) in (A) ST362 *unc-7(e139) ncIs53(myo-3p*::*Arch*::*gfp*, *rig-3p*::*mCherry)* and (B) ST361 *unc-31(e169) ncIs53(myo-3p*::*Arch*::*gfp*, *rig-3p*::*mCherry)* animals.
For comparison, modulation of the pumping rate in ST322 *ncIs53* and ST324 *unc-7(e139); ncIs53* animals (A), and in ST322 *ncIs53* and ST361 *unc-31(e169); ncIs53* animals (B) is also shown.
(EPS)

S5 Fig Laser ablation of I1 neurons. (A1) Expression pattern of mCherry in an ST322 *ncIs53(myo-3p*::*Arch*::*gfp*, *rig-3p*::*mCherry)* animal at the L2 stage. White arrowheads indicate bilateral I1 neurons. The scale bar represents 20 μm.
(A2) Expression pattern of mCherry after laser ablation of both I1 neurons in the worm shown in (A1). No mCherry signal was observed at the position indicated by the white arrowheads. The scale bar represents 20 μm.
(B) A fluorescent micrograph of the I1-ablated animal shown in (A1) and (A2) at the adult stage. The scale bar represents 50 μm.
(EPS)

S6 Fig Modulation of the pumping rate by illumination with green light at an intensity of 5.5 mW/mm2 in animals carrying the integrated transgene shown in Fig 5. (EPS)

S1 Table Statistical data for Fig 2: Modulation of pharyngeal pumping caused by silencing of the body wall muscles from illumination with green light (22 mW/mm2) in ST300: *Ex(myo-3p::Arch::gfp, rol-6d)* animals carrying mutations in genes involved in neurotransmission. The data were analyzed using the Steel's many-one rank sum test.
(XLSX)

S2 Table Statistical data for Fig 4: Comparison of modulation of the pumping rate among mutant animals carrying *ncIs53(myo-3p::Arch::gfp, rig-3p::mCherry)*. The data were analyzed using the Kruskal-Wallis test with post-hoc Steel-Dwass multiple comparison test
(XLSX)

S3 Table Statistical data for Fig 5: Statistical data for Fig 5: Effects of ablation of I1 neurons on modulation of the pumping rate with green light (5.5 mW/mm2) in animals carrying *ncIs53(myo-3p::Arch::gfp, rig-3p::mCherry)*. The data were analyzed using the Mann-Whiteny test.
(XLSX)
